# Interrelationship of Multiple Endothelial Dysfunction Biomarkers with Chronic Kidney Disease

**DOI:** 10.1371/journal.pone.0132047

**Published:** 2015-07-01

**Authors:** Jing Chen, L. Lee Hamm, Emile R. Mohler, Alhakam Hudaihed, Robin Arora, Chung-Shiuan Chen, Yanxi Liu, Grace Browne, Katherine T. Mills, Myra A. Kleinpeter, Eric E. Simon, Nader Rifai, Michael J. Klag, Jiang He

**Affiliations:** 1 Department of Medicine, Tulane University School of Medicine, New Orleans, Louisiana, United States of America; 2 Tulane Hypertension and Renal Center of Excellence, Tulane University School of Medicine, New Orleans, Louisiana, United States of America; 3 Department of Epidemiology, Tulane University School of Public Health and Tropical Medicine, New Orleans, Louisiana, United States of America; 4 Department of Medicine, University of Pennsylvania School of Medicine, Philadelphia, Pennsylvania, United States of America; 5 Department of Pathology, Children's Hospital, Harvard Medical School, Boston, Massachusetts, United States of America; 6 The Johns Hopkins Bloomberg School of Public Health, Baltimore, Maryland, United States of America; University of Florida, UNITED STATES

## Abstract

The interrelationship of multiple endothelial biomarkers and chronic kidney disease (CKD) has not been well studied. We measured asymmetric dimethylarginine (ADMA), L-arginine, soluble intercellular adhesion molecule-1 (sICAM-1), soluble vascular adhesion molecule-1 (sVCAM-1), soluble E-selectin (sE-selectin), von Willebrand factor (vWF), flow-mediated dilation (FMD), and nitroglycerin-induced dilation (NID) in 201 patients with CKD and 201 community-based controls without CKD. Multivariable analyses were used to examine the interrelationship of endothelial biomarkers with CKD. The multivariable-adjusted medians (interquartile ranges) were 0.54 (0.40, 0.75) in patients with CKD vs. 0.25 (0.22, 0.27) μmol /L in controls without CKD (p<0.0001 for group difference) for ADMA; 67.0 (49.6, 86.7) vs. 31.0 (27.7, 34.2) μmol/L (p<0.0001) for L-arginine; 230.0 (171.6, 278.6) vs. 223.9 (178.0, 270.6) ng/mL (p=0.55) for sICAM-1; 981.7 (782.6, 1216.8) vs. 633.2 (507.8, 764.3) ng/mL (p<0.0001) for sVCAM-1; 47.9 (35.0, 62.5) vs. 37.0 (28.9, 48.0) ng/mL (p=0.01) for sE-selectin; 1320 (1044, 1664) vs. 1083 (756, 1359) mU/mL (p=0.008) for vWF; 5.74 (3.29, 8.72) vs. 8.80 (6.50, 11.39)% (p=0.01) for FMD; and 15.2 (13.5, 16.9) vs. 19.1 (17.2, 21.0)% (p=0.0002) for NID, respectively. In addition, the severity of CKD was positively associated with ADMA, L-arginine, sVCAM-1, sE-selectin, and vWF and inversely associated with FMD and NID. Furthermore, FMD and NID were significantly and inversely correlated with ADMA, L-arginine, sVCAM-1, sE-selectin, and vWF. In conclusion, these data indicate that multiple dysfunctions of the endothelium were present among patients with CKD. Interventional studies are warranted to test the effects of treatment of endothelial dysfunction on CKD.

## Introduction

Chronic kidney disease (CKD) is highly prevalent worldwide and a major risk factor for end-stage renal disease (ESRD), cardiovascular disease (CVD), and premature death [1–3]. Endothelial dysfunction plays a critical role in the development of atherosclerosis and vascular lesions, which might be a shared common pathogenic pathway for CKD and CVD [4,5]. However, the interrelationship of multiple dysfunctions of endothelium with CKD is not well studied.

Endothelial dysfunction, measured by impaired endothelium-dependent flow-mediated dilation (FMD) and circulating biomarkers, has been observed among patients with CKD [6–14]. For instance, Van Guldener et al. reported impaired FMD in hemodialysis and peritoneal dialysis patients [6,7]. Impaired FMD was also associated with proteinuria in patients with diabetes or hypertension [8,9]. In addition, plasma asymmetric dimethylarginine (ADMA), an endogenous nitric oxide synthase (NOS) inhibitor, was found to be associated with CKD progression [10,11]. Other endothelial dysfunction biomarkers in the inflammation and thrombosis pathways were also reported to be associated with decreased kidney function [12,13]. However, the associations between the biomarkers of endothelial dysfunction and CKD were inconsistent among studies [6–14]. In addition, important confounding factors were not adjusted in many studies. Furthermore, there are no studies examining the interrelationship of multiple endothelial dysfunction biomarkers with CKD. The objectives of this study are to investigate the association of multiple biomarkers of endothelial dysfunction with the risk and severity of CKD, as well as to examine the correlation among these biomarkers in patients with CKD.

## Subjects and Methods

### Study participants

We recruited 201 patients with CKD and 201 controls without CKD in the greater New Orleans area from 2007 to 2010. CKD patients aged 21–74 years were recruited from nephrology and internal medicine clinics via physicians’ referral by trained research staff in the study area. All eligible CKD patients identified in the recruiting clinics were invited to participate in the study. CKD was defined as estimated glomerular filtration rate (eGFR) <60 ml/min/1.73 m^2^ or presence of albuminuria (≥30 mg/24-hours). Patients were excluded if they had a history of chronic dialysis, kidney transplants, immunotherapy in the past six months, chemotherapy within the past two years, or current clinical trial participation that may have an impact on CKD. Additional exclusion criteria were history of HIV or AIDS and inability or unwillingness to give informed consent. Controls were recruited through mass mailing to residents aged 21–74 years living in the same area according to zip code. The eligibility of controls was assessed by a clinic screening visit. Individuals were included if they had no evidence of CKD (eGFR >60 ml/min/1.73 m^2^ and no persistent albuminuria).

Tulane University Institutional Review Board approved this study, and written informed consent was obtained at the screening visit from all study participants.

### Measurements

A standard questionnaire was administered by trained staff at a clinical visit to obtain demographic information, lifestyle risk factors (including cigarette smoking, alcohol drinking, and physical activity), and self-reported history of CVD, diabetes, hypercholesterolemia, and hypertension, as well as the use of antihypertensive, lipid-lowering, and anti-diabetic medications and aspirin.

Three blood pressure (BP) measurements were obtained at a clinical visit by trained and certified staff according to a common protocol adapted from procedures recommended by the American Heart Association [15]. A standard mercury sphygmomanometer was used, and one of four cuff sizes (pediatric, regular adult, large, or thigh) was chosen based on participant arm circumference. BP was measured with the participant in the sitting position after they had rested for 5 minutes. Body height and weight were measured twice with the participant in light indoor clothing without shoes during their clinical visit and were used to calculate body mass index (BMI).

An overnight fasting blood sample was collected to measure blood biomarkers of endothelial dysfunction, plasma glucose, serum creatinine and cholesterol, and triglycerides. eGFR was estimated from serum creatinine, sex, age, and race using the CKD-EPI equation [16]. A 24-hour urinary sample was collected to measure creatinine and albumin excretion. Serum cholesterol and triglyceride levels were assayed using an enzymatic procedure on the Hitachi 902 automatic analyzer (Roche Diagnostics, Indianapolis, IN, USA). Serum glucose was measured using a hexokinase enzymatic method (Roche Diagnostics, Indianapolis, IN, USA). Serum creatinine was measured using the Roche enzymatic method (Roche—Hitachi P-Module instrument with Roche Creatininase Plus assay, Hoffman-La Roche, Basel, Switzerland). Urinary concentrations of albumin and creatinine were measured with a DCA 2000 Analyzer (Bayer AG, Leverkusen, Germany).

Serum soluble intercellular adhesion molecule-1 (sICAM-1), soluble vascular adhesion molecule-1 (sVCAM-1), and soluble E-selectin (sE-selectin) were measured by an ELISA assay using the quantitative sandwich enzyme immunoassay technique (R&D Systems, Minneapolis, MN). Day-to-day variability of the assay was 6.0 to 10.1% for sICAM-1, 8.5 to 10.2% for sVCAM-1, and 5.7 to 8.8% for sE-selectin. Plasma von Willebrand factor (vWF) was measured by an ELISA assay (American Diagnostica, Greenwich, CT) with a day-to-day variability less than 10.0%. Plasma ADMA and L-arginine were determined by HPLC according to a standard protocol with a coefficient of variation of 3.5% for ADMA and 2.1% for L-arginine [17]. All laboratory measures were conducted at the Department of Pathology of Boston Children's Hospital.

Endothelium-dependent FMD in response to reactive hyperemia and endothelium-independent, nitroglycerin-induced vasodilation (NID) were measured using high resolution ultrasound (Siemens Acuson Ultrasound System, San Jose, CA) during the clinical examination [18]. Participants were examined in the supine position after 15 minutes of rest and after at least 10-hours of fasting. In addition, participants were requested to avoid alcohol, cigarette smoking, coffee/tea, and exercise for at least 10 hours before their FMD measurement. B-mode scans of the right brachial artery were obtained in longitudinal sections 2 to 8 cm above the elbow by use of a 7.5-MHz linear array transducer. After baseline images were obtained, a blood pressure (BP) cuff was inflated to 50 mm Hg above the participant’s systolic BP for 5 minutes. Digitized images of the right brachial artery diameter were captured continuously for 1 minute before application of the cuff and for 5 minutes after cuff deflation. A second baseline measurement was taken after 5 minutes of recovery. Subsequently, sublingual nitroglycerin (0.4 mg) was administered, and brachial artery measurements were obtained after 5 minutes as described above. Ultrasound images were recorded on a super VHS videocassette for offline electronic image analysis with the use of Image Pro Software (Image Pro, Data Translation Inc, Marlboro, Mass) at the Vascular Laboratory of the University of Pennsylvania. Endothelium-dependent FMD was calculated as the maximal percentage change in vessel size during hyperemia. Endothelium-independent NID was calculated as the percentage change in vessel size from baseline to 5 minutes after administration of sublingual nitroglycerin.

### Statistical analyses

Medians and interquartile ranges of endothelial dysfunction biomarkers were calculated for CKD patients and controls, and the Mann-Whitney test was used to test differences in the unadjusted medians [19]. Quantile regression was used to obtain adjusted medians (interquartile ranges), and the Wald test was used to test differences in the adjusted medians between CKD patients and controls [20]. Age, gender, race, high-school education, current cigarette smoking, weekly alcohol consumption, physical activity (≥ twice per week), BMI, low-density lipoprotein (LDL) cholesterol, serum glucose, systolic BP, history of CVD, and medication use were adjusted in these analyses. Multivariable linear regression was used to examine the association of eGFR and urinary albumin with biomarkers after adjustment for the previously mentioned covariates. Log transformations were used for all biomarkers, except NID, because they are not normally distributed. Multivariate-adjusted regression coefficients are reported for a one standard deviation increase in biomarkers. All analyses were performed using SAS version 9.2 statistical software (Cary, NC).

## Results

The general characteristics of study participants by CKD status are presented in Table 1. Those with CKD were older, less educated, and less likely to drink alcohol compared to those without CKD. In addition, they were more likely to have a history of CVD, hypertension, diabetes, and hypercholesterolemia and to have been taking medications for these conditions. Mean BMI, systolic BP, serum glucose, and urinary albumin were significantly higher, while LDL-cholesterol and eGFR were lower, in CKD patients compared to controls.

**Table 1:** Characteristics of 201 Patients with Chronic Kidney Disease and 201 Controls without Chronic Kidney Disease.

Variables	CKD patients (n = 201)	Non-CKD controls (n = 201)	P-value
Age, years	55.9 ± 9.9	52.5 ± 10.0	0.0007
Male, %	55.2	45.3	0.05
Blacks, %	60.7	51.2	0.06
High-school education, %	58.5	81.6	<0.0001
Current cigarette smoking, %	53.7	48.8	0.32
Weekly alcohol drinking, %	27.9	59.2	<0.0001
History of cardiovascular disease, %	43.7	7.0	<0.0001
History of hypertension, %	88.1	23.9	<0.0001
History of diabetes, %	49.3	5.5	<0.0001
History of hypercholesterolemia, %	65.7	30.9	<0.0001
Use of antihypertensive agents, %	79.6	15.4	<0.0001
Use of hypoglycemic agents, %	34.3	3.0	<0.0001
Use of lipid lowering agents, %	21.9	9.0	0.0003
Use of aspirin, %	34.8	8.5	<0.0001
Body-mass index, kg/m^2^	32.2 ± 7.8	28.9 ± 6.4	<0.0001
Systolic blood pressure, mm Hg	132.2 ± 21.0	122.0 ± 14.7	<0.0001
Diastolic blood pressure, mm Hg	77.2 ± 13.5	77.6 ± 9.4	0.77
Plasma glucose, mg/dL	119.9 ± 46.7	103.4 ± 35.4	<0.0001
LDL-cholesterol, mg/dL	103.3 ± 45.9	118.2 ± 30.2	<0.0001
Serum creatinine, mg/dL	2.0 ± 1.1	0.9 ± 0.2	<0.0001
eGFR, mL/min/1.73 m^2^	43.3 ± 19.9	96.7 ± 16.8	<0.0001
Urinary albumin, mg/24 hrs	74.5 (12.3, 417.4)	5.9 (4.1, 11.4)	<0.0001

LDL = low-density lipoprotein; eGFR = estimated glomerular filtration rate.

Table 2 shows the adjusted medians or means of endothelial dysfunction biomarkers by CKD status. After adjustment for potential CKD risk factors, including the use of antihypertensive agents, hypoglycemic agents, lipid-lowering agents, and aspirin, the medians of all blood endothelial biomarkers, except sICAM-1, were significantly higher in CKD patients compared to controls. The multiple-adjusted medians of FMD and means of NID were significantly lower in CKD patients compared to controls.

**Table 2:** Biomarkers of Endothelial Dysfunction According to Chronic Kidney Disease Status.

	Age-gender-race-adjusted median (IQR)			Multivariable-adjusted median (IQR)*		
	CKD patients (n = 201)	Non-CKD controls (n = 201)	*P* for difference	CKD patients (n = 201)	Non-CKD controls (n = 201)	*P* for difference
ADMA, μmol/L	0.51 (0.39, 0.72)	0.25 (0.22, 0.27)	<0.0001	0.54 (0.40, 0.75)	0.25 (0.22, 0.27)	<0.0001
L-Arginine, μmol/L	61.9 (48.8, 87.5)	30.9 (27.5, 34.4)	<0.0001	67.0 (49.6, 86.7)	31.0 (27.7, 34.2)	<0.0001
sICAM-1, ng/mL	225.0 (170.5, 277.9)	216.8 (178.0, 269.5)	0.33	230.0 (171.6, 278.6)	223.9 (178.0, 270.6)	0.55
sVCAM-1, ng/mL	967.8 (773.9, 1202.9)	619.0 (501.8, 752.9)	<0.0001	981.7 (782.6, 1216.8)	633.2 (507.8, 764.3)	<0.0001
sE-selectin, ng/mL	47.5 (34.7, 63.8)	37.1 (26.7, 49.1)	0.0001	47.9 (35.0, 62.5)	37.0 (28.9, 48.0)	0.010
vWF, mU/mL	1370 (1053, 1668)	1034 (735, 1319)	<0.0001	1320 (1044, 1664)	1083 (756, 1359)	0.008
FMD, %	5.58 (2.74, 8.84)	8.40 (6.39, 11.4)	<0.0001	5.74 (3.29, 8.72)	8.80 (6.50, 11.4)	0.014
NID,^†^ %	14.6 (13.4, 15.7)	20.0 (18.8, 21.1)	<0.0001	15.2 (13.5, 16.9)	19.1 (17.2, 21.0)	0.0002

IQR = inter-quartile range; ADMA = asymmetric dimethylarginine; sICAM-1 = soluble intercellular adhesion molecule-1; sVCAM-1 = soluble vascular adhesion molecule-1; sE-selectin = soluble E-selectin; vWF = von Willebrand factor; FMD = flow-mediated dilation; and NID = nitroglycerin-induced dilation.

*Adjusted for age, gender, race, high-school education, physical activity, current cigarette smoking, weekly alcohol drinking, body mass index, LDL-cholesterol, plasma glucose, systolic blood pressure, history of cardiovascular disease, and use of antihypertensive agents, hypoglycemic agents, lipid lowering agents, and aspirin.

^†^ Mean (95% confidence intervals).

Table 3 presents age-gender-race-adjusted and multivariate-adjusted regression coefficients (95% confidence intervals) of one standard deviation higher log-transformed endothelial dysfunction measures and NID with eGFR and log-transformed urinary albumin excretion. In the multivariate-adjusted models, higher log (ADMA), log (L-arginine), and log (sVCAM-1) were significantly associated with a decreased eGFR and increased albuminuria, while higher log (sE-selectin) was only associated with increased albuminuria, and log (vWF) was only associated with a decreased eGFR. A higher log (FMD) and NID were significantly associated with an increased eGFR and decreased albuminuria.

**Table 3:** Multivariable-Adjusted Regression Coefficients (95% Confidence Intervals) of One Standard Deviation Higher Log-transformed Endothelial Dysfunction Biomarkers with Estimated-GFR and Log-transformed Urinary Albumin Excretion.

	eGFR, mL/min/1.73 m^2^		Log-albuminuria, mg/24-hours	
	Effect size (95% CI)	p-value	Effect size (95% CI)	p-value
Log (ADMA, 0.5μmol/L)				
Age-gender-race-adjusted	-31.0 (-32.1, -29.9)	<0.0001	1.37 (1.21, 1.52)	<0.0001
Multivariable-adjusted*	-29.6 (-30.9, -28.28)	<0.0001	1.18 (1.01, 1.36)	<0.0001
Log (L-arginine, 0.5 μmol/L)				
Age-gender-race-adjusted	-30.0 (-31.3, -28.7)	<0.0001	1.35 (1.19, 1.5)	<0.0001
Multivariable-adjusted*	-27.9 (-29.4, -26.3)	<0.0001	1.15 (0.98, 1.32)	<0.0001
Log (sICAM-1 0.4 ng/mL)				
Age-gender-race-adjusted	1.90 (-1.24, 5.04)	0.23	-0.02 (-0.22, 0.18)	0.86
Multivariable-adjusted*	3.31 (0.66, 5.97)	0.01	-0.13 (-0.3, 0.05)	0.16
Log (sVCAM-1, 0.4 ng/mL)				
Age-gender-race-adjusted	-17.6 (-20.27, -14.94)	<0.0001	1.0 (0.82, 1.17)	<0.0001
Multivariable-adjusted*	-12.42 (-15.17, -9.68)	<0.0001	0.69 (0.51, 0.87)	<0.0001
Log (sE-selectin, 0.5 ng/mL)				
Age-gender-race-adjusted	-5.13 (-8.32, -1.93)	0.002	0.58 (0.38, 0.77)	<0.0001
Multivariable-adjusted*	-2.11 (-5.0, 0.78)	0.15	0.34 (0.16, 0.53)	0.0004
Log (vWF, 0.4 mU/mL)				
Age-gender-race-adjusted	-7.21 (-10.35, -4.07)	<0.0001	0.3 (0.09, 0.51)	0.005
Multivariable-adjusted*	-3.2, (-6.0, -0.41)	0.02	0.06 (-0.13, 0.24)	0.56
Log (FMD, 0.8%)				
Age-gender-race-adjusted	7.95 (4.73, 11.17)	<0.0001	-0.55 (-0.75,-0.36)	<0.0001
Multivariable-adjusted*	4.53 (1.60, 7.46)	0.003	-0.28 (-0.48, -0.09)	0.004
NID, 9.0%				
Age-gender-race-adjusted	10.9 (7.41, 14.38)	<0.0001	-0.62 (-0.85, -0.38)	<0.0001
Multivariable-adjusted*	7.15 (3.75, 10.54)	<0.0001	-0.27 (-0.5,-0.04)	0.02

ADMA = asymmetric dimethylarginine; sICAM-1 = soluble intercellular adhesion molecule-1; sVCAM-1 = soluble vascular adhesion molecule-1; sE-selectin = soluble E-selectin; vWF = von Willebrand factor; FMD = flow-mediated dilation; and NID = nitroglycerin-induced dilation.

*Adjusted for age, gender, race, high-school education, physical activity, current cigarette smoking, weekly alcohol drinking, body mass index, LDL-cholesterol, plasma glucose, systolic blood pressure, history of cardiovascular disease, and use of antihypertensive agents, hypoglycemic agents, lipid lowering agents, and aspirin.

Correlation coefficients among endothelial dysfunction biomarkers are presented in Table 4. FMD and NID were highly correlated with each other, and they were significantly and inversely correlated with circulating levels of log (ADMA), log (L-arginine), log (sVCAM-1), log (sE-selectin), and log (vWF). Circulating log (ADMA) was highly correlated with circulating levels of log (L-arginine) and log (sVCAM-1) and moderately correlated with log (sE-selectin) and log (vWF). Circulating levels of log (sICAM-1), log (sVCAM-1), log (sE-selectin), and log (vWF) were significantly and positively correlated with each other.

**Table 4:** Pearson Correlation Coefficients (*P* values) Among Endothelial Function Biomarkers.

	Log (FMD)	Log (ADMA)	Log (L arginine)	Log (sICAM-1)	Log (sVCAM-1)	Log (sE-selectin)	Log (vWF)
Log (ADMA)	-0.335 (<0.0001)						
Log (L-arginine)	-0.320 (<0.0001)	0.974 (<0.0001)					
Log (sICAM-1)	0.000 (0.998)	-0.086 (0.092)	-0.063 (0.217)				
Log (sVCAM-1)	-0.150 (0.003)	0.507 (<0.0001)	0.513 (<0.0001)	0.306 (<0.0001)			
Log (sE-selectin)	-0.177 (0.0005)	0.257 (<0.0001)	0.240 (<0.0001)	0.183 (0.0003)	0.242 (<0.0001)		
Log (vWF)	-0.137 (0.007)	0.254 (<0.0001)	0.242 (<0.0001)	0.107 (0.035)	0.327 (<0.0001)	0.085 (0.092)	
NID	0.521 (<0.0001)	-0.366 (<0.0001)	-0.355 (<0.0001)	-0.035 (0.520)	-0.172 (0.0013)	-0.178 (0.0009)	-0.133 (0.013)

ADMA = asymmetric dimethylarginine; sICAM-1 = soluble intercellular adhesion molecule-1; sVCAM-1 = soluble vascular adhesion molecule-1; sE-selectin = soluble E-selectin; vWF = von Willebrand factor; FMD = flow-mediated dilation; and NID = nitroglycerin-induced dilation.

In addition, inflammatory biomarkers, log-tumor necrosis factor-alpha (r = –0.174, p<0.001), and interleukin-6 (r = –0.104, p = 0.04) were significantly and inversely correlated with FMD. However, log-C reactive protein was not significantly correlated with FMD (r = –0.048, p = 0.35).

### Sensitivity analysis

After excluding participants with systolic BP ≥140/90 mm Hg or fasting plasma glucose ≥126 mg/dL, the multivariable-adjusted medians (inter-quartile ranges) were 0.53 (0.39, 0.76) vs. 0.24 (0.22,0.27) μmol/L for ADMA (p<0.0001), 65.2 (50.3, 91.8) vs. 29.6 (27.2, 33.4) μmol/L for L-arginine (p<0.0001), 929.6 (711.6, 1136.7) vs. 595.0 (499.8, 773.0) ng/mL for sVCAM-1 (p<0.0001), and 6.1 (3.5, 9.2) vs. 9.1 (6.9, 12.2) % for FMD (p = 0.03) in patients with CKD (n = 91) and controls (n = 157), respectively.

## Discussion

The present study indicated that abnormalities in multiple endothelial pathways, i.e., the nitric oxide pathway (ADMA and L-arginine), inflammation (sVCAM-1 and sE-selectin), thrombosis (vWF), and impaired endothelial dependent dilation (FMD), were significantly and independently associated with CKD. These associations remained after adjustment for established CKD risk factors as well as the use of antihypertensive, antidiabetic, and lipid-lowering medications and aspirin. In addition, these endothelial dysfunction biomarkers were associated with CKD severity measured by eGFR and/or albuminuria. Furthermore, multiple endothelial dysfunction biomarkers were correlated with each other. These findings suggest that endothelial dysfunction in multiple pathways is present in patients with CKD.

These study findings may have important clinical and public health implications. CKD is a common public health problem and is associated with increased risk of CVD [1–3]. Previous studies have reported that endothelial dysfunction is a risk factor for CVD in the general population, as well as in patients with CKD [4,21,22]. Our study added new information on multiple endothelial biomarker abnormalities associated with increased risk of CKD. The strengths of this study include that multiple circulating biomarkers of endothelial dysfunction and endothelium-dependent FMD, as well as endothelium-independent NID, were measured simultaneously and that established risk factors for CKD were adjusted in multivariable analyses. However, this cross-sectional analysis cannot establish temporal relationships between endothelial biomarkers and risk of CKD. Future longitudinal cohort studies and clinical trials are warranted to test the causal relationship of endothelial dysfunction on CKD development and progression.

Our study is the first to examine the correlation of brachial artery FMD with multiple circulating biomarkers of endothelial dysfunction among patients with CKD. The noninvasive brachial artery FMD is a valid, widely-used clinical measure of endothelium-dependent vasomotor function in humans, which reflects nitric oxide (NO) availability [23]. Our study indicated that FMD was highly significantly correlated with plasma ADMA and L-arginine, two circulating biomarkers of NO bioavailability. In addition, our study found that FMD was moderately correlated with endothelium-mediated pro-inflammatory and pro-thrombotic molecules, including sVCAM-1, sE-selectin, and vWF, which suggests that NO deficiency may co-exist with other pathogenesis of endothelial dysfunction in patients with CKD.

Impaired brachial artery FMD was associated with increased risk of CVD in several prospective cohort studies in the general population [24–26]. In addition, previous studies also reported impaired FMD in ESRD patients on dialysis [6,7]. Our study indicated that impaired brachial artery FMD was present in early-stage CKD patients prior to dialysis. Our study also indicated that FMD was associated with the severity of CKD measured by eGFR and albuminuria. These findings are consistent with previous reports that albuminuria was associated with impaired FMD in patients with diabetes or hypertension [8,9]. In addition, our study findings suggest that vasodilation induced by nitroglycerin (an endogenous NO donor) may be impaired in patients with CKD.

ADMA is an endogenous inhibitor of NOS, and increased levels of ADMA may be related to reduced NO bioavailability and endothelial dysfunction [27]. Two clinical studies have examined the association between plasma ADMA level and the progression of CKD [10,11]. Ravani et al. reported that plasma ADMA was an independent risk factor for ESRD and mortality in 131 patients with CKD in Italy [10]. Fliser et al. also found that plasma ADMA was associated with progression of mild and moderate CKD in 227 non-diabetic young kidney disease patients [11]. Our study supports these findings and showed a strong and independent association of ADMA with both eGFR and albuminuria. Our study is one of the first investigations that found an elevated level of plasma L-arginine in pre-dialysis CKD patients. The underutilization of L-arginine due to NOS inhibited by ADMA or decreased excretion of L-arginine might contribute to elevated levels of L-arginine in CKD patients. This observation might partially explain why L-arginine supplementation did not improve kidney function in CKD patients [28].

Cell adhesion molecules, such as sICAM-1, sVCAM-1, and sE-selectin, trigger leukocyte homing, adhesion, and migration into the subendothelial space, processes fundamental to the formation of atherosclerotic lesions [29]. Elevated circulating levels of sICAM-1, sVCAM-1 and sE-selectin were found in ESRD patients on hemodialysis in some studies but not in others [12,13,30,31]. Our study indicated that serum levels of sVCAM-1 and sE-selectin were significantly increased in CKD patients. vWF is released into circulation by activated endothelial cells and mediates platelet adhesion to injured endothelium, the first step in thrombus formation [32]. vWF was found to be higher in CKD patients compared to normal controls [33,34]. Our study provides additional evidence that vWF is independently associated with worse eGFR.

In conclusion, our study indicates that multiple dysfunctions of endothelium were present among patients with CKD. In addition, multiple biomarkers of endothelial dysfunction were correlated with each other. Our data warrant future interventional studies to test the effects of treatment of endothelial dysfunction on the development and progression of CKD.

## References

[1] El Nahas AM, Bello AK. Chronic kidney disease: the global challenge. Lancet. 2005;365: 331–40. 15664230 10.1016/S0140-6736(05)17789-7

[2] Muntner P, He J, Hamm L, Loria C, Whelton PK. Renal insufficiency and subsequent death resulting from cardiovascular disease in the United States. J Am Soc Nephrol. 2002;13: 745–753. 11856780 10.1681/ASN.V133745

[3] Go AS, Chertow GM, Fan D, McCulloch CE, Hsu CY. Chronic kidney disease and the risks of death, cardiovascular events, and hospitalization. N Engl J Med. 2004;351: 1296–305. 15385656 10.1056/NEJMoa041031

[4] Fliser D, Wiecek A, Suleymanlar G, Ortiz A, Massy Z, Lindholm B, et al The dysfunctional endothelium in CKD and in cardiovascular disease: mapping the origin(s) of cardiovascular problems in CKD and of kidney disease in cardiovascular conditions for a research agenda. Kidney Int Suppl. 2011;1: 6–9. 10.1038/kisup.2011.6 PMC4089605 25018895

[5] Hogas SM, Voroneanu L, Serban DN, Segall L, Hogas MM, Serban IL, et al Methods and potential biomarkers for the evaluation of endothelial dysfunction in chronic kidney disease: a critical approach. J Am Soc Hypertens. 2010;4: 116–27. 10.1016/j.jash.2010.03.008 20470996

[6] Van Guldener C, Lambert J, Janssen MJ, Donker AJ, Stehouwer CD. Endothelium-dependent vasodilatation and distensibility of large arteries in chronic haemodialysis patients. Nephrol Dialy Transplant. 1997;12 Suppl 2: 14–8. 9269693

[7] Van Guldener C, Janssen MJ, Lambert J, Steyn M, Donker AJ, Stehouwer CD. Endothelium-dependent vasodilatation is impaired in peritoneal dialysis patients. Nephrol Dialy Transplant. 1998;13: 1782–6. 10.1093/ndt/13.7.1782 9681728

[8] Stehouwer CD, Henry RM, Dekker JM, Nijpels G, Heine RJ, Bouter LM., et al Microalbuminuria is associated with impaired brachial artery, flow-mediated vasodilation in elderly individuals without and with diabetes: further evidence for a link between microalbuminuria and endothelial dysfunction—the Hoorn Study. Kidney Int. 2004;92: S42–4. 10.1111/j.1523-1755.2004.09211.x 15485416

[9] Malik AR, Sultan S, Turner ST, Kullo IJ. Urinary albumin excretion is associated with impaired flow- and nitroglycerin-mediated brachial artery dilatation in hypertensive adults. J Human Hypertens. 2007;21: 231–8. 17230233 10.1038/sj.jhh.1002143

[10] Ravani P, Tripepi G, Malberti F, Testa S, Mallamaci F, Zoccali C, et al Asymmetrical dimethylarginine predicts progression to dialysis and death in patients with chronic kidney disease: a competing risks modeling approach. J Am Soc Nephrol. 2005;16: 2449–55. 15944335 10.1681/ASN.2005010076

[11] Fliser D, Kronenberg F, Kielstein JT, Morath C, Bode-Boger SM, Haller H, et al Asymmetric dimethylarginine and progression of chronic kidney disease: the mild to moderate kidney disease study. J Am Soc Nephrol. 2005;6: 2456–61. 10.1681/ASN.2005020179 15930091

[12] Stam F, van Guldener C, Schalkwijk CG, ter Wee PM, Donker AJ, Stehouwer CD. Impaired renal function is associated with markers of endothelial dysfunction and increased inflammatory activity. Nephrol Dial Transplant. 2003;18: 892–8. 12686661 10.1093/ndt/gfg080

[13] Upadhyay A, Larson MG, Guo CY, Vasan RS, Lipinska I, O'Donnell CJ et al Inflammation, kidney function and albuminuria in the Framingham Offspring cohort. Nephrol Dial Transplant. 2011;26: 920–6. 10.1093/ndt/gfq471 20682604 PMC3108344

[14] Stam F, van Guldener C, Becker A, Dekker JM, Heine RJ, Bouter LM, et al Endothelial dysfunction contributes to renal function-associated cardiovascular mortality in a population with mild renal insufficiency: the Hoorn study. J Am Soc Nephrol. 2006;17: 537–45. 16382015 10.1681/ASN.2005080834

[15] Pickering TG, Hall JE, Appel LJ, Falkner BE, Graves J, Hill MN, et al Recommendations for blood pressure measurement in humans and experimental animals: part 1: blood pressure measurement in humans. Circulation. 2005;111: 697–716. 15699287 10.1161/01.CIR.0000154900.76284.F6

[16] Levey AS, Coresh J, Greene T, Lesley AS, Zhang Y, Hendriksen S, et al Using standardized serum creatinine values in the modification of diet in renal disease study equation for estimating glomerular filtration rate. Ann Intern Med. 2006;145: 247–254. 16908915 10.7326/0003-4819-145-4-200608150-00004

[17] Teerlink T, Nijveldt RJ, de Jong S, van Leeuwen PA. Determination of arginine, asymmetric dimethylarginine, and symmetric dimethylarginine in human plasma and other biological samples by high-performance liquid chromatography. Analytical Biochemistry. 2002;303: 131–7. 11950212 10.1006/abio.2001.5575

[18] Lekakis J, Abraham P, Balbarini A, Blann A, Boulanger CM, Cockcroft J, et al Methods for evaluating endothelial function: a position statement from the European Society of Cardiology Working Group on Peripheral Circulation. Eur J Cardiovasc Prev Rehabil. 2011;18: 775–89. 10.1177/1741826711398179 21450600

[19] LaVange LM, Koch GG. Rank score tests. Circulation. 2006;114: 2528–33. 17146004 10.1161/CIRCULATIONAHA.106.613638

[20] McGreevy KM, Lipsitz SR, Linder JA, Rimm E, Hoel DG. Using median regression to obtain adjusted estimates of central tendency for skewed laboratory and epidemiologic data. Clin Chem. 2009;55: 165–169. 10.1373/clinchem.2008.106260 19028825

[21] Böger RH, Sullivan LM, Schwedhelm E, Wang TJ, Maas R, Benjamin EJ, et al Plasma asymmetric dimethylarginine and incidence of cardiovascular disease and death in the community. Circulation. 2009;119: 1592–600. 10.1161/CIRCULATIONAHA.108.838268 19289633 PMC2742491

[22] Tripepi G, Mattace Raso F, Sijbrands E, Seck MS, Maas R, Boger R, et al Inflammation and asymmetric dimethylarginine for predicting death and cardiovascular events in ESRD patients. Clin J Am Soc Nephrol. 2011;6: 1714–21. 10.2215/CJN.11291210 21642364

[23] Green DJ, Dawson EA, Groenewoud HM, Jones H, Thijssen DH. Is flow-mediated dilation nitric oxide mediated? A meta-analysis. Hypertension. 2014;63: 376–82. 10.1161/HYPERTENSIONAHA.113.02044 24277765

[24] Inaba Y, Chen JA, Bergmann SR. Prediction of future cardiovascular outcomes by flow-mediated vasodilatation of brachial artery: a meta-analysis. Int J Cardiovasc Imaging. 2010;26: 631–640. 10.1007/s10554-010-9616-1 20339920

[25] Yeboah J, Crouse JR, Hsu FC, Burke GL, Herrington DM. Brachial flow-mediated dilation predicts incident cardiovascular events in older adults: the Cardiovascular Health Study. Circulation. 2007;115: 2390–7. 17452608 10.1161/CIRCULATIONAHA.106.678276

[26] Yeboah J, Folsom AR, Burke GL, Johnson C, Polak JF, Post W, et al Predictive value of brachial flow-mediated dilation for incident cardiovascular events in a population-based study: the multi-ethnic study of atherosclerosis. Circulation. 2009;120: 502–509. 10.1161/CIRCULATIONAHA.109.864801 19635967 PMC2740975

[27] Kielsteina JT, Zoccali C. Asymmetric dimethylarginine: a novel marker of risk and a potential target for therapy in chronic kidney disease. Curr Opin Nephrol Hypertens. 2008;17: 609–615. 10.1097/MNH.0b013e328314b6ca 18941355

[28] Cherla G, Jaimes EA. Role of L-arginine in the pathogenesis and treatment of renal disease. J Nutr. 2004;134(10 Suppl): 2801S–2806S. 15465789 10.1093/jn/134.10.2801S

[29] Deanfield JE, Halcox JP, Rabelink TJ. Endothelial function and dysfunction: testing and clinical relevance. Circulation. 2007;15: 1285–95. 10.1161/CIRCULATIONAHA.106.652859 17353456

[30] Papayianni A, Alexopoulos E, Giamalis P, Gionanlis L, Belechri AM, Koukoudis P, et al Circulating levels of ICAM-1, VCAM-1, and MCP-1 are increased in haemodialysis patients: association with inflammation, dyslipidemia, and vascular events. Nephrol Dial Transplant. 2002;17: 435–41. 11865089 10.1093/ndt/17.3.435

[31] Bonomini M, Reale M, Santarelli P, Stuard S, Settefrati N, Albertazzi A. Serum levels of soluble adhesion molecules in chronic renal failure and dialysis patients. Nephron. 1998;79: 399–407. 9689154 10.1159/000045084

[32] Mannucci PM. Von Willebrand factor: a marker of endothelial damage? Arterioscler Thromb Vasc Biol. 1998;18: 1359–62. 9743222 10.1161/01.atv.18.9.1359

[33] Landray MJ, Wheeler DC, Lip GY, Newman DJ, Blann AD, McGlynn FJ, et al Inflammation, endothelial dysfunction, and platelet activation in patients with chronic kidney disease: the chronic renal impairment in Birmingham (CRIB) study. Am J Kidney Dis. 2004;43: 244–53. 14750089 10.1053/j.ajkd.2003.10.037

[34] Bash LD, Erlinger TP, Coresh J, Marsh-Manzi J, Folsom AR, Astor BC. Inflammation, hemostasis, and the risk of kidney function decline in the Atherosclerosis Risk in Communities (ARIC) Study. Am J Kidney Dis. 2009;53: 596–605. 10.1053/j.ajkd.2008.10.044 19110358 PMC2778194

